# Phytochemical Profile and Selective Anticancer Activity of *Parietaria judaica* L. Extracts

**DOI:** 10.3390/molecules30132739

**Published:** 2025-06-25

**Authors:** Izabela Bielecka, Dorota Natorska-Chomicka, Wioleta Dołomisiewicz, Arlindo Rodrigues Fortes, Katarzyna Dos Santos Szewczyk

**Affiliations:** 1 Department of Pharmaceutical Botany, Medical University of Lublin, 20-093 Lublin, Poland; bieleckaizabela33@gmail.com (I.B.); wioleta.dolomisiewicz@umlub.pl (W.D.); 2 Chair and Department of Toxicology, Faculty of Pharmacy, Medical University of Lublin, 20-090 Lublin, Poland; dorota.natorska-chomicka@umlub.pl; 3 Escola Superior de Ciências Agrárias e Ambientais, Universidade de Cabo Verde, Praia CP 84, Cape Verde; arlinfortes@gmail.com

**Keywords:** *Parietaria judaica*, Urticeae, LC-MS, anticancer, cytotoxicity, polyphenols, alfavaca

## Abstract

*Parietaria judaica* L. (alfavaca-de-cobra) was investigated as a potential source of anticancer compounds. Leaf extracts obtained using solvents of different polarities were evaluated for their phytochemical profiles and cytotoxic activities against a panel of human cancer cell lines (glioblastoma LN-229, lung NCI-H1563, breast MDA-MB-231, liver HepG2, renal 769-P, cervical HeLa, and melanoma A-375) and a noncancerous HEK-293 cell line. LC-ESI-MS/MS analysis confirmed that the extracts are rich in polyphenols, including phenolic acids and flavonoids. Cytotoxicity was assessed via MTT and SRB assays, demonstrating dose-dependent antiproliferative effects. Among the extracts, the ethanolic fraction (PJ-E) exhibited the strongest cytotoxicity, with an IC_50_ of 11.82 µg/mL against HeLa cells, while displaying a significantly higher IC_50_ (139.42 µg/mL) against HEK-293, indicating tumor selectivity. The water extract (PJ-W) showed selective activity against lung cancer cells (IC_50_ = 87.69 µg/mL), with minimal toxicity toward normal cells. The methanol/acetone extract (PJ-M) displayed intermediate activity, whereas the hexane extract (PJ-H) was the least effective. These findings highlight *P. judaica*, particularly its ethanolic extract, as a promising source of natural anticancer agents. Further research focusing on the isolation of active constituents, formulation development, and in vivo validation is warranted to support its therapeutic potential.

## 1. Introduction

Natural products have long been a cornerstone in cancer drug discovery, with over half of the current anticancer drugs being derived from or inspired by compounds from natural sources [1]. This reflects the immense chemical diversity and bioactivity of plant secondary metabolites, which can modulate key pathways in carcinogenesis. In the search for new anticancer agents, medicinal plants used in traditional medicine offer promising leads as their ethnopharmacological history suggests therapeutic potential.

*Parietaria judaica* L. (F. Urticaceae), commonly known as alfavaca-de-cobra, is a perennial herb native to Europe and the Mediterranean region. The aerial parts (leafy stems) of *P. judaica* are traditionally used in Cabo Verde in the form of herbal teas and infusions for the treatment of hemorrhoids, abdominal pain, genital infections, edema, cystitis/urinary tract infections, diabetes, and hair loss [2,3]. Folk medicine also attributes anti-inflammatory and wound-healing properties to this plant, using it for conditions like cystitis, rheumatism, skin ulcers, and burns. In fact, *P. judaica* and its close relative *P. officinalis* (pellitory-of-the-wall) were historically regarded as therapeutically interchangeable, reflecting similar traditional uses and constituents [4]. Despite its rich ethnobotanical background, *P. judaica* has been largely overlooked in modern pharmacological research, and its potential anticancer properties remain underexplored.

In addition to their ethnomedicinal uses, *P. judaica* and related *Parietaria* species have been reported to exhibit other biological activities. Recent studies indicate that *P. judaica* extracts possess notable antioxidant and antimicrobial properties in vitro [5,6], supporting some of the traditional uses of this herb.

Phytochemical studies indicate that *P. judaica* is abundant in phenolic compounds, particularly flavonoids. A recent chemotaxonomic review of Urticaceae found *Parietaria* to be one of the genera richest in *O*-glycosylated and *O*-methylated flavonols [7]. These include quercetin and kaempferol glycosides (e.g., rutin, quercitrin, isoquercitrin) and phenolic acids such as gallic and ferulic acid, as is also confirmed by our analytical data. Such polyphenols are known for broad bioactivity profiles, including antioxidant and cytotoxic effects. Notably, many flavonoids can interfere with cancer cell proliferation and survival pathways; for instance, quercetin has demonstrated pro-apoptotic and antiproliferative effects in various tumor models [1]. The presence of these bioactive constituents in *P. judaica* provides a strong rationale for investigating its anticancer potential.

In this study, we present a focused investigation on *P. judaica* leaf extracts as anticancer agents. The aim of the paper was to characterize the phytochemical composition of different solvent extracts and evaluate their cytotoxic activity against a panel of cancer cell lines, in order to identify the most effective extract and most sensitive cancer targets. By including a noncancerous cell line in our assays, we also assessed the selectivity of *P. judaica* extracts for cancer cells over normal cells. Given the gap in the literature and the lack of prior reports on the anticancer effects of *P. judaica*, this work provides novel insights into alfavaca bioactivity and lays the groundwork for the future development of natural product-based anticancer therapies from this traditionally valued herb.

## 2. Results and Discussion

### 2.1. Phytochemical Composition and Implications for Activity

**Figure 1:**
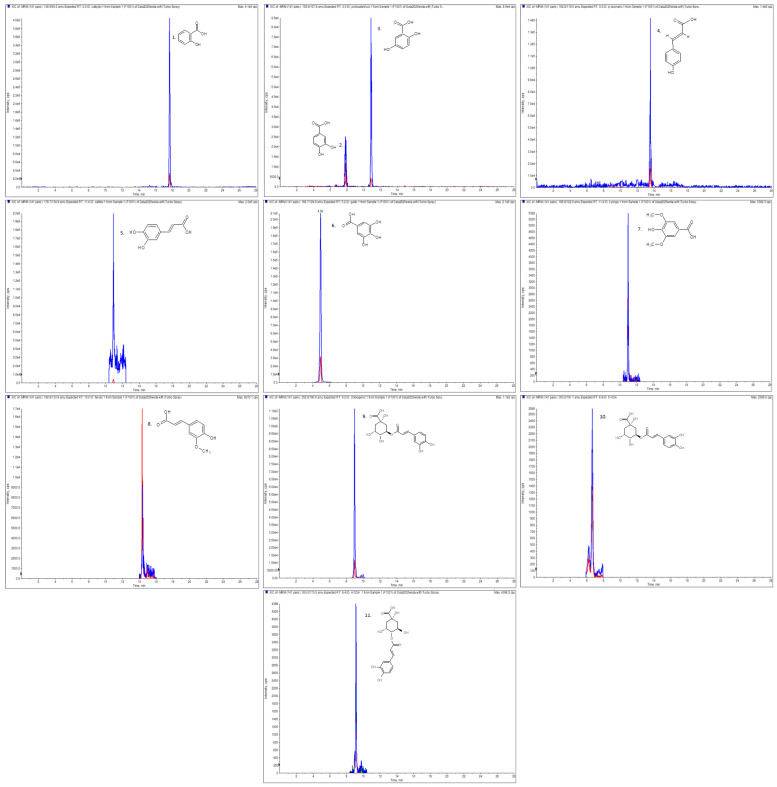
The chromatogram in MRM mode of phenolic acids in PJ-H: (1) salicylic acid; (2) protocatechuic acid; (3) gentisic acid; (4) *p*-coumaric acid; (5) caffeic acid; (6) gallic acid; (7) syringic acid; (8) ferulic acid; (9) chlorogenic acid; (10) neochlorogenic acid; (11) cryptochlorogenic acid.

The first stage of our study involved the qualitative and quantitative analysis of flavonoids and phenolic acids in the crude extracts obtained from the leaves of *P. judaica*. The chemical profile of *P. judaica* leaf extracts revealed a complex mixture of phenolic compounds that is possibly responsible for the observed bioactivities. Using an optimized LC-ESI-MS/MS procedure, we identified the highest number of phenolic compounds in the 70% ethanolic extract (33 compounds), and 32 compounds in the methanol/acetone/water (3:1:1) extract. Representative chromatograms illustrating the presence of phenolic acids in the hexane extract of the leaves of *P. judaica* (PJ-H) are presented in Figure 1. The concentrations of individual compounds, quantified by comparing the peak areas to the calibration curves prepared from appropriate standards, are detailed in Table 1.

In our analysis, a hexane extract of *P. judaica* (PJ-H) yielded exceptionally high levels of gallic acid (855.29 ± 45.76 µg/g DE) and flavonoid glycosides [notably quercetin derivatives like quercitrin (3194.64 ± 124.26 µg/g DE) and isoquercetin (850.67 ± 32.69 µg/g DE)], whereas the water (PJ-W) and 70% ethanol (PJ-E) extracts contained slightly lower total phenolic contents but were enriched in specific compounds. The water extract had abundant ferulic acid (1216.27 ± 23.85 µg/g DE) and quercitrin (391.69 ± 18.56 µg/g DE), and PJ-E contained large amounts of isoquercetin (3143.42 ± 77.77 µg/g DE), rutin (1003.08 ± 68.44 µg/g DE), and astragalin (932.69 ± 24.89 µg/g DE).

Quercitrin (391.69–3194.64 µg/g DE), isoquercetin (347.45–3143.42 µg/g DE), rutin (119.97–1249.60 µg/g DE), astragalin (11.09–1235.14 µg/g DE), ferulic acid (698.68–1216.27 µg/g dry extract), and gallic acid (14.13–855.29 µg/g DE) occurred in the highest amount.

To our best knowledge, the literature mainly shows information concerning the total phenol, flavonoid, tannin, saponin, or carbohydrate content rather than a detailed chemical composition of *Parietaria* species [5,8,9]. The only work on the identification of flavonoids concerns *P. officinalis*. Budzianowki et al. (1985) identified 3-glucosides and 3-rutinosides of quercetin, kaempferol and isorhamnetin, 3-sophorosides of quercetin and kaempferol, and 3-neohesperidosides of kaempferol and isorhamnetin in the leaves and flowers of *P. officinalis* [10].

However, some of the compounds we identified in *P. judaica* leaf were previously detected in the Urticaceae family (Table 2).

Our findings align with the known phytochemistry of the genus *Parietaria*, which is characterized by a high degree of flavonoid glycosylation [7]. Such glycosides can contribute to bioactivity, either directly or via in vivo deglycosylation to aglycones. The presence of quercetin is particularly noteworthy as this flavonoid is a well-documented anticancer agent that can induce apoptosis and sensitize tumor cells to chemotherapy by modulating the PI3K/AKT/mTOR pathway [1]. Similarly, ferulic acid—found in high concentration in the water extract (PJ-W)—is reported to reverse multidrug resistance in cancer cells by inhibiting the PI3K/AKT/NF-κB signaling axis [1]. These compounds may act synergistically, inducing the observed cytotoxic effects, which suggests a multifaceted mechanism characteristic of complex herbal extracts.

### 2.2. Differential Cytotoxicity of Extracts and Cancer Cell Line Sensitivity

All *P. judaica* extracts tested demonstrated cytotoxic activity against cancer cells but with marked differences in potency and selectivity corresponding to their phytochemical composition. The results for all extracts in the MTT (tetrazolium dye) and SRB (sulforhodamine B) assays are presented in Table 3 and Table 4. The ethanolic extract (PJ-E) stood out as the most active, reaching IC_50_ values below 20 µg/mL in several cell lines after 24 h exposure. In particular, PJ-E was highly effective against HeLa cervical carcinoma cells (IC_50_ = 11.82 µg/mL at 24 h), suggesting a rapid induction of cell death in this line. Melanoma A-375 cells were also very sensitive to PJ-E upon longer exposure (48 h), with an IC_50_ = 8.29 µg/mL, indicating that this extract exerts strong cytostatic/cytotoxic effects on malignant cells of diverse tissue origins. The higher efficacy of PJ-E can be related to its broad spectrum of polyphenols. Indeed, the 70% ethanol solvent extracted a wide array of flavonoids and phenolic acids from *P. judaica*. Notably, PJ-E contained exceptionally high levels of certain phenolics (e.g., isoquercetin, rutin, astragalin), which may contribute significantly to its potent activity. These constituents may act additively or synergistically to kill cancer cells.

In contrast, the methanol/acetone/water extract (PJ-M)—despite a comparably high total phenolic content—was less active than PJ-E, suggesting that the presence of specific highly bioactive compounds in PJ-E (and not the total amount of phenolics itself) is a key factor in its higher efficacy. The hexane extract (PJ-H) was comparatively weak in anticancer activity. Its IC_50_ values exceeded 100 µg/mL for most cancer cell lines at 24 h, indicating only mild cytotoxicity even at high concentrations. PJ-H did show a weak effect on HepG2 hepatocellular carcinoma cells (IC_50_ = 107.86 µg/mL) and A-375 melanoma cells (IC_50_ = 113.68 µg/mL) at 24 h, but overall, it was the least potent extract. This is not surprising given that PJ-H, being non-polar, contains mainly lipophilic constituents (e.g., fatty acids, hydrocarbons) and very few polar phenolics; thus, it lacks the rich flavonoid content associated with anti-tumor effects. Accordingly, the low activity of PJ-H indicates that the anticancer effects of *P. judaica* are primarily attributed to its polar constituents.

Interestingly, the water extract (PJ-W) and the methanol/acetone/water extract (PJ-M) exhibited intermediate profiles, demonstrating a certain degree of tumor selectivity. PJ-W was particularly effective against the NCI-H1563 lung cancer cells (24 h; IC_50_ = 87.69 µg/mL) while being much less toxic to normal HEK-293 cells (IC_50_ = 253.35 µg/mL). This suggests that PJ-W contains compounds selectively active against lung carcinoma. One possible explanation is the high content of coumaric acid and ferulic acid in PJ-W; while these phenolics are mild cytotoxins on their own, they might target pathways more crucial in certain cancer cell types (such as those related to oxidative stress or metabolism in lung cancer cells) [31,32,33]. PJ-M, on the other hand, showed its strongest effects on HeLa and LN-229 (glioblastoma) cells (IC_50_ = 63.21 µg/mL and = 74.18 µg/mL, respectively, at 24 h), while also saving the HEK-293 line (IC_50_ = 202.34 µg/mL). The PJ-M extract probably contained a wide mixture of polar compounds due to the combination of solvents. Its activity (with that against cervical and brain tumor cells being the most sensitive) may reflect the influence of certain flavonoids or phenolic acids that have selectivity for those cancer types. For instance, caffeic acid and quercetin glycosides (present in PJ-M) have been reported to induce apoptosis preferentially in some carcinoma cells but not in rapidly proliferating normal cells [31,34,35].

The differential sensitivities observed among the cancer cell lines provide insight into potential mechanisms. HeLa cervical cancer cells were consistently among the most sensitive to *P. judaica* extracts (especially PJ-E and PJ-M). This could be related to their p53-deficient status which often makes them reliant on alternative survival pathways that polyphenols can disrupt. Likewise, A-375 melanoma cells responded dramatically to PJ-E (notable in the 48 h assay), which might be due to flavonoids triggering oxidative stress or mitotic arrest in these highly metabolic cells. In contrast, MDA-MB-231 triple-negative breast cancer cells showed moderate sensitivity, requiring higher extract doses for inhibition (PJ-E IC_50_ = 49.15 µg/mL at 48 h), possibly because these cells are mesenchymal-like and drug-resistant; even so, *P. judaica* extracts did inhibit their growth. The renal carcinoma 769-P line and HepG2 liver cancer were also moderately affected, but interestingly, PJ-H and PJ-W had some of their lowest IC_50_ values in HepG2 (IC_50_ = 81.89 µg/mL for PJ-H, IC_50_ = 83.46 µg/mL for PJ-W at 48 h), hinting that certain constituents (perhaps ferulic acid in PJ-W, or lipid-soluble components in PJ-H) might interfere with liver cancer cell viability. Overall, the broad activity of *P. judaica* extracts against multiple tumor cell types suggests a multi-mechanistic cytotoxic action, likely involving the induction of apoptosis and cell cycle arrest, as is common with many polyphenol-rich extracts [34].

### 2.3. Selectivity Toward Cancer Cells and Comparison with Other Natural Products

A critical aspect of any potential anticancer agent is selectivity—the ability to cause the death of cancer cells more easily than normal cells [36]. In this study, the inclusion of HEK-293 (human embryonic kidney) cells as a non-tumorigenic control allowed us to measure the therapeutic window of *P. judaica* extracts. Encouragingly, all extracts showed some degree of selectivity, and notably, PJ-E and PJ-M exhibited large IC_50_ differences between cancer and normal cells. PJ-E was about 10–12 times more toxic to HeLa cancer cells than to HEK-293, and PJ-M had a similar selectivity factor (~3-fold more toxic to HeLa than HEK-293, and ~2.7-fold for LN-229 vs. HEK-293). PJ-W was extremely non-toxic to HEK-293 (no growth inhibition up to the highest concentration tested), yet it was able to inhibit certain cancers, which suggests a high therapeutic index for that extract in particular. PJ-H, while the least potent against cancers, also had practically no effect on normal cells (HEK-293 viability remained >90% even at 300 µg/mL of PJ-H), emphasizing that the cytotoxic compounds of *P. judaica* are in its polar fractions.

These findings of cancer-selective action align with reports on other Urticaceae plant extracts and polyphenol-rich natural products. For example, *Urtica dioica* leaf extract has demonstrated the capacity to reduce breast cancer cell viability while sparing normal fibroblasts [34,37]. The selective cytotoxicity of *U. dioica* has been attributed to its flavonoid content (such as kaempferol, quercetin, and rutin) inducing apoptosis in cancer cells through mitochondrial pathways and cell cycle arrest, with minimal impact on non-cancerous cells [34,37]. *P. judaica* has a similar phytochemical composition rich in flavonoids and phenolic acids, which possibly act on common cellular targets—for instance, triggering pro-apoptotic proteins (Bax, caspases) in cancer cells that have abnormal survival signaling. The fact that *P. judaica* extracts exhibited greater cytotoxicity toward rapidly dividing cancer cells than toward non-transformed renal cells indicates a certain degree of tumor specificity, likely associated with differences in cellular uptake or metabolism of phytochemicals, or with a higher basal level of oxidative stress in cancer cells, making them more susceptible to further oxidative damage induced by polyphenols.

Compared to other natural product extracts studied for anticancer properties, *P. judaica* performs on par with many known anti-tumor herbs. Its ethanol extract achieved an IC_50_ of less than 10 µg/mL against melanoma cells, which is comparable to potent plant-derived compounds like curcumin [38] or epigallocatechin gallate (EGCG) [39] under similar conditions. Moreover, the multi-extract approach in this study illustrates how different solvent fractions can differ in their in efficacy: often, ethanol or hydroalcoholic extracts achieve the best balance of extracting active polar compounds, a trend also observed, for example, for extracts of *Curcuma* spp. [40,41], *Camellia sinensis* (L.) Kuntze [42,43], or *Vitex* species [44,45]. Few previous studies have examined *Parietaria* species for anticancer activity [5,46]. Our results thus fill a knowledge gap and suggest that *P. judaica* has the anticancer potential found in other flavonoid-rich medicinal plants. Overall, the demonstration of selective anticancer effects by *P. judaica* extracts reinforces the concept that traditional medicinal herbs may contain potent anti-tumor agents. The pattern of activity seen here (e.g., potent effects on cervical and melanoma cells) could guide the choice of cancer models for further study of *P. judaica* and suggests that some tumors (perhaps those of epithelial origin) might be particularly sensitive to its constituents.

## 3. Materials and Methods

### 3.1. Chemicals and Reagents

Reference substances were supplied by Sigma-Aldrich Fine Chemicals (St. Louis, MO, USA), while acetonitrile, formic acid, and water were supplied for LC analysis by Merck (Darmstadt, Germany). All other chemicals were of analytical grade and were obtained from the Polish Chemical Reagent Company (POCH, Gliwice, Poland).

### 3.2. Plant Material

The leaves of *Parietaria judaica* L. were collected in Paúl, Santo Antão, Cape Verde in July 2023 during flowering season. Taxonomical identification was confirmed by Prof. Arlindo Rodrigues Fortes. The voucher specimen was deposited in the Escola Superior de Ciências Agrárias e Ambientais (PI-0723).

### 3.3. Preparation of the Extracts

The collected plant material was air-dried at an average temperature of 24.0 ± 0.5 °C in the shade to constant weight and then pulverized. Next, 15.00 g of dried *P. judaica* leaf powder was extracted with hexane (PJ-H), a mixture of methanol, acetone, and water (3:1:1; *v*/*v*/*v*; PJ-M); ethanol 70% (*v*/*v*) (PJ-E); and distilled water (PJ-W) (3 × 75 mL with each solvent). Each extraction was performed by sonication in an ultrasonic bath at a controlled temperature of 45 ± 2 °C for 30 min. The extracts thus obtained were filtered and combined. The combined filtrates from each solvent were concentrated under reduced pressure (rotary evaporation) and frozen, then lyophilized using a vacuum concentrator (Free Zone 1 apparatus; Labconco, Kansas City, KS, USA) to yield dried crude extracts. The yields of the extracts were as follows: PJ-H—0.19 g; PJ-M—1.65 g; PJ-E—3.05 g; and PJ-W—3.32 g. Extraction methods and conditions were chosen to maximize phenolic yields, and indeed the yields differed by solvent. Each lyophilized extract was reconstituted in HPLC-grade methanol (10 mg/mL) and filtered (0.22 µm) before LC-MS/MS injection.

### 3.4. LC-ESI-MS/MS Analysis of Phenolic Acids and Flavonoids

The phenolic and flavonoid compounds were quantified using high-performance liquid chromatography coupled with electrospray ionization tandem mass spectrometry (LC-ESI-MS/MS), using a slightly modified method previously described by Nowacka et al. [47] and Pietrzak et al. [48]. An Agilent 1200 Series HPLC system (Agilent Technologies, Santa Clara, CA, USA) connected to a 3200 QTRAP Mass spectrometer (AB Sciex, Framingham, MA, USA) with electrospray ionization source (ESI) operating in negative-ion mode. Analyses were used for all analytes. Both were controlled with Analyst 1.5 software (AB Sciex, Framingham, MA, USA), which was also used for data interpretation.

Chromatographic separation of phenolic acids, flavonoid aglycones, and flavonoid glycosides was carried out at 25 °C on a Zorbax SB-C18 column (2.1 × 150 mm, 1.8 µm particle size; Agilent Technologies, Santa Clara, CA, USA). The mobile phase consisted of 0.1% aqueous formic acid (solvent A) and acetonitrile with 0.1% formic acid (solvent B). The injection volume was 3 µL and the flow rate was 300 µL/min. The gradient was changed as follows: 0–2 min—20% B; 3–4 min—25% B; 5–6 min—35%; 8–12 min—65% B; 14–16 min—80% B; 20–28 min—20% B.

The ESI-MS analysis was performed in negative ionization mode under the following conditions: a capillary temperature of 450 °C, curtain gas at 30 psi, nebulizer gas at 50 psi, and a source voltage of −4500 V. Triplicate injections were made for each standard solution and sample. The limits of detection (LOD) and quantification (LOQ) for all analytes were determined at a signal-to-noise ratio of 3:1 and 10:1, respectively. Qualitative identification of compounds was performed by the comparison of MS/MS spectra and LC retention time with the corresponding standards tested under the same conditions. The calibration curves obtained in MRM mode were used for quantification of analytes. Detailed conditions of LC-MS analysis are given in Tables S1–S4.

### 3.5. Reagents and Cell Culture

Eagle’s Minimum Essential Medium (EMEM), Dulbecco’s Modification of Eagle’s Medium (DMEM), RPMI-1640 Medium, F-12K Medium, and Fetal Bovine Serum (FBS) were purchased from Corning (Manassas, VA, USA). DPBS and all antibiotics were obtained from PAN-Biotech GmbH (Aidenbach, Germany). The MTT assay was obtained from Invitrogen (Waltham, MA, USA). DMSO (dimethyl sulfoxide) was obtained from POCH (Gliwice, Poland). The Sulforhodamine B (SRB), trichloroacetic acid (TCA) and Tris base solution (pH 10.5) were purchased from Sigma-Aldrich (St. Louis, MO, USA).

The present study was performed using tumor cell cultures supplied by the American Type Culture Collection (ATCC), including LN-229 (human glioblastoma), NCI-H1563 (human non-small cell lung cancer adenocarcinoma), MDA-MB-231 (human mammary gland adenocarcinoma), HepG2 (human liver cancer), 769-P (human renal cell adenocarcinoma), HeLa (human cervical adenocarcinoma), A-375 (human melanoma), and HEK-293 (human kidney embryonic cells; Merck^®^ 85120602) which was used as a normal reference cell line. The cells were cultured in a humidified incubator at 37 °C and 5% CO_2_ atmosphere. The culture medium—EMEM (HeLa, HepG2), DMEM (HEK-293, LN-229, A-375), RPMI-1640 (NCI-H1563, 769-P), and F12K (MDA-MB-231)—was supplemented with 10% fetal bovine serum and 100 U/mL penicillin, 100 μg/mL streptomycin, and 2.5 μg/mL amphotericin B (PAN-Biotech GmbH, Aidenbach, Germany).

### 3.6. Cell Viability Assay

The cytotoxicity was evaluated with MTT assay (European Centre for the Validation of Alternative Methods, Database Service on Alternative Methods to Animal Experimentation). Cell viability was determined by a mitochondria-dependent reaction based on the ability of viable cells to the transformation of tetrazolium salts MTT into purple formazan by mitochondrial dehydrogenases. The cells were seeded into 96-well plates in a volume of 200 µL per well at the density of 1 × 10^5^ cells/mL. The extracts were dissolved in DMSO and subsequently diluted to the required concentration with the respective cell culture medium. The solutions were prepared ex tempore. The cells were exposed to various concentrations (10, 50, 100, 150, and 200 µg/mL) of the tested extracts for 24 h at 37 °C. After incubation, 20 µL of MTT (5 mg/mL) stock in PBS was added to each well and incubated for 3 h at 37 °C. In the next step, the culture medium was removed, and the crystals of formazan were dissolved in 100 µL of DMSO (dimethylsulfoxide). The absorbance of each well was measured at 550 nm using a PowerWave™ automated absorbance microplate reader (BioTek Instruments, Inc., Winooski, VT, USA). Each experiment was performed in triplicate with three replicates for each concentration. Based on the MTT assay results, the IC_50_ values of the tested extracts were determined from the concentration–response curves. The final concentration of DMSO did not exceed 0.1% *v*/*v*.

### 3.7. The SRB Assay

The Sulforhodamine B (SRB) assay was employed to evaluate the cytotoxic activity of the tested extracts on the selected cell lines [49,50]. The basis of this colorimetric method is the determination of the total protein content in the tested sample, which is directly proportional to the number of cells. For the assay, cells were initially seeded into a 96-well microplate at a density of 5000 to 10,000 cells per well, depending on the specific growth characteristics of the cell line used. The cells were adhered by incubating the plate at 37 °C in a humidified atmosphere with 5% CO_2_ for 24 h. Following the initial incubation period, the cells were exposed to the test compounds at varying concentrations. The extracts were prepared analogously to the MTT test by dissolving them in a suitable medium. Next, the treated cells were incubated with the compounds for a further 48 h. After the treatment period, the cells were fixed by carefully removing the medium from each well and adding 25 μL of ice-cold trichloroacetic acid (TCA) directly to the cells. The plate was incubated at 4 °C for one hour to ensure thorough fixation of cellular proteins. Following fixation, the wells were washed five times with distilled water to remove residual TCA and any unbound material, and the plate was allowed to air-dry at room temperature. Once the plate was completely dry, the cells were stained by adding 50 μL of 0.4% SRB solution (prepared in 1% acetic acid) to each well. The plate was then incubated at room temperature for 30 min, allowing the SRB dye to bind stoichiometrically to the basic amino acids of cellular proteins. After staining, the excess dye was removed by washing the wells five times with 1% acetic acid, and the plate was again allowed to air-dry completely. To solubilize the protein-bound SRB dye, 100 μL of 10 mM Tris base solution (pH 10.5) was added to each well, and the plate was gently shaken for 5 min to ensure complete solubilization of the dye. The absorbance of each well was then measured at 490 nm using a microplate reader. The optical density at this wavelength is directly proportional to the total protein content and, consequently, the number of viable cells remaining after treatment. The cytotoxicity of each compound was expressed as the percentage of viable cells relative to the untreated controls, and IC_50_ values (the concentration required to inhibit 50% of cell growth) were determined from dose–response curves.

## 4. Conclusions and Future Perspectives

In conclusion, *Parietaria judaica* L. seems to be a noteworthy source of natural compounds with anticancer activity. Its leaf extracts—particularly the ethanol-derived fraction (PJ-E)—exhibited potent cytotoxic effects against a range of human cancer cell lines, while largely sparing non-cancerous cells. This selective toxicity is a desirable attribute for anticancer agents and is possibly attributable to *P. judaica* high polyphenol content (flavonoids and phenolic acids), and it positions this plant as a strong candidate for further anticancer development.

At the same time, the differential sensitivities observed among the cancer cell lines in our study provide insight into potential mechanisms. The rapid onset of cell death (within 24 h) caused by PJ-E in HeLa cells indicates apoptosis rather than merely cytostasis. Flavonoids such as quercetin and luteolin are known to activate intrinsic apoptotic pathways by causing mitochondrial membrane depolarization and caspase activation in cancer cells [34]. Our observation that HeLa and A-375 cells—which responded to low extract concentrations—likely underwent apoptosis is consistent with such a mechanism. In contrast, the moderate activity in some lines at 24 h that improved by 48 h (as seen for MDA-MB-231 or HepG2 with PJ-E) suggests cell cycle arrest may precede cell death in those cases. Many polyphenols can induce a halt in G_0_/G_1_ or G_2_/M phases in cancer cells, often by modulating cyclin-dependent kinases and checkpoints. For instance, *U. dioica* extract was reported to cause G_0_/G_1_ arrest in breast cancer cells by inhibiting PI3K/AKT signaling [34]; by analogy, *P. judaica* extracts may have similar effects on cell cycle regulators in our tested cell lines.

Thus, our future work will focus on isolating the key bioactive compounds and elucidating their mechanisms of action—whether through inducing apoptosis, cell cycle arrest, or other pathways—in the cancer cell types identified as the most sensitive. In particular, detailed mechanism-of-action studies (for example, using flow cytometry to detect cell-cycle arrest and annexin V staining to confirm apoptosis) will be valuable to determine how *P. judaica* extracts induce cancer cell death. Bioactivity-guided fractionation of the extracts is justified to determine the compounds responsible for the cytotoxic effects. It is possible that minor constituents or synergistic combinations of compounds are contributing to the activity. Our results suggest a multi-pathway action characteristic of herbal extracts, in which one compound may block survival pathways or drug-efflux pumps, while others induce oxidative stress or DNA damage.

Additionally, the in vitro results reported here will need to be validated in vivo. Crucially, studies in animal models of cancer are needed to evaluate the therapeutic efficacy and safety of *P. judaica* extracts or their major compounds, as factors like bioavailability and metabolism (not captured in cell culture) could influence performance. We have also identified certain limitations in our study: for example, we used a single “normal” cell line (HEK-293) as a control, which, while offering some insight into selectivity, does not fully represent the spectrum of normal human tissues. Including additional non-malignant cells (e.g., primary human fibroblasts or epithelial cells) in future tests would provide a more comprehensive toxicity profile.

From a pharmaceutical development perspective, formulating the extracts or isolated compounds for improved delivery (e.g., as nanoparticles or liposomal formulations) could increase their stability and bioavailability, addressing potential pharmacokinetic issues. Combination therapies might also be explored: the polyphenols from *P. judaica* could be used together with conventional chemotherapeutics to potentiate their effects or overcome resistance. For instance, ferulic acid has a known ability to inhibit P-glycoprotein and NF-κB, thereby sensitizing cancer cells to other drugs [1]. Such integrative approaches, supported by our findings of synergistic interactions within the extracts, may improve treatment results.

Moreover, given the complex mixture nature of the extracts, there is an opportunity to explore combination treatments: *P. judaica* polyphenols might be used together with conventional chemotherapeutic drugs to potentiate their effects or mitigate resistance, a strategy supported by the ability of compounds such as ferulic acid to inhibit drug resistance mechanism [1].

In summary, *P. judaica* is emerging as a promising source of anticancer agents. Combining traditional knowledge and modern bioassay-guided research, this plant may yield new anticancer drug candidates or adjunct therapies in the quest for more effective and safer cancer treatments [1]. The present study lays a strong foundation for these prospects and underscores the value of exploring under-investigated medicinal plants for anticancer properties.

## Figures and Tables

**Table 1:** Content [µg/g DE] of phenolic acids, flavonoid aglycones, and flavonoid glycosides determined by LC-ESI-MS/MS in *Parietaria judaica* leaf *extracts* (Average, n ≥ 3). PJ-H—hexane, PJ-M—methanol/acetone/water (3:1:1, *v*/*v*/*v*), PJ-E—70% ethanol, PJ-W—water. Abbreviations: <LOQ—a concentration below the limit of quantification—the metabolite was detected, but its concentration could not be determined; nd—not detected; DE—dry extract.

Compound	PJ-H	PJ-M	PJ-E	PJ-W
**Phenolic Acids [µg/g DE]**				
Gallic acid	855.29 ± 45.76	165.14 ± 1.36	52.47 ± 0.62	14.13 ± 0.32
3-*O*-caffeoylquinic acid (neochlorogenic acid)	15.88 ± 0.82	1.86 ± 0.05	0.36 ± 0.03	0.98 ± 0.04
Protocatechuic acid	58.02 ± 0.98	75.97 ± 1.59	59.83 ± 1.24	197.71 ± 4.51
5-caffeoylquinic acid (chlorogenic acid)	284.56 ± 5.56	77.10 ± 4.09	33.90 ± 1.21	14.79 ± 0.08
4-caffeoylquinic acid (cryptochlorogenic acid	4.83 ± 0.29	1.15 ± 0.15	<LOQ	<LOQ
Gentisic acid	<LOQ	<LOQ	<LOQ	<LOQ
Caffeic acid	83.68 ± 1.31	69.55 ± 1.59	72.15 ± 2.49	83.02 ± 2.92
Syringic acid	nd	21.23 ± 0.68	7.94 ± 0.34	49.28 ± 1.32
Vanilic acid	nd	nd	nd	22.54 ± 1.14
p-Coumaric acid	5.73 ± 0.45	42.24 ± 0.23	64.67 ± 1.24	228.45 ± 11.39
Ferulic acid	nd	698.68 ± 29.53	<LOQ	1216.27 ± 23.85
Rosmarinic acid	nd	nd	<LOQ	nd
Salicylic acid	<LOQ	39.82 ± 2.29	<LOQ	<LOQ
**Flavonoid aglycones [µg/g dry extract]**				
Catechin	<LOQ	nd	nd	nd
EGCG	nd	<LOQ	4.63 ± 0.21	nd
Dihydromyricetin	nd	5.86 ± 0.20	6.29 ± 0.25	nd
Myricetin	<LOQ	<LOQ	<LOQ	nd
Eriodictyol	<LOQ	<LOQ	<LOQ	<LOQ
Luteolin	<LOQ	<LOQ	<LOQ	<LOQ
Quercetin	21.12 ± 0.13	20.22 ± 1.34	7.46 ± 0.59	13.76 ± 0.53
3-*O*-Methylquercetin	<LOQ	nd	<LOQ	nd
Apigenin	<LOQ	<LOQ	<LOQ	<LOQ
Kaempferol	<LOQ	<LOQ	<LOQ	<LOQ
Isorhamnetin	<LOQ	<LOQ	<LOQ	<LOQ
**Flavonoid glycosides [µg/g dry extract]**				
Luteolin 3’,7’-diglucoside	nd	<LOQ	nd	nd
Quercetin-3-*O*-rutinoside (Rutin)	119.97 ± 3.60	1249.60 ± 4.54	1003.08 ± 68.44	215.14 ± 0.10
Apigenin—6-*C*-glucoside (Isovitexin)	5.012 ± 0.16	5.38 ± 0.11	71.27 ± 3.11	9.56 ± 1.70
Luteolin-7-*O*-glucoside (Luteoloside)	18.84 ± 0.95	<LOQ	<LOQ	47.98 ± 2.65
Quercetin-3-*O*-glucoside (Isoquercetin)	850.67 ± 32.69	2942.50 ± 95.40	3143.42 ± 77.77	347.45 ± 11.66
Eriodictyol-7-*O*-glucopyranoside	nd	<LOQ	<LOQ	nd
Kaempferol—3-*O*-rutinoside (Nicotiflorin)	nd	252.17 ± 8.63	166.52 ± 7.16	<LOQ
Isorhamnetin-3-*O*-rutinoside (Narcissoside)	15.33 ± 0.03	78.22 ± 3.41	53.45 ± 0.93	19.53 ± 0.37
Kaempferol—3-*O*-glucoside (Astragalin)	266.99 ± 8.17	1235.14 ± 34.07	932.69 ± 24.89	11.09 ± 0.32
Isorhamnetin-3-glucoside	<LOQ	124.00 ± 2.73	115.48 ± 7.16	nd
Quercetin 3-*O*-rhamnoside (Quercitrin)	3194.64 ± 124.26	581.43 ± 13.63	706.11 ±03.11	391.69 ± 18.56
Apigenin 7-*O*-glucoside (Apigetrin, Cosmosiin)	<LOQ	nd	nd	<LOQ
Naringenin 7-*O*-glucoside	11.03 ± 0.75	4.26 ± 0.57	4.73 ± 0.53	nd

**Table 2:** Compounds identified in *P. judaica* leaf extracts that were detected in the Urticaceae family.

Compound	Species	Part of the Plant	Reference(s)
Ferulic acid	*Urtica dioica*	leaves	[11,12]
Gallic acid			
Gentisic acid			
Syringic acid			
Caffeic acid	*Urtica dioica*	leaves	[11]
	*Urtica artichocaulis* Hand.-Mazz	aerial parts	[12]
Chlorogenic acid	*Urtica artichocaulis*	aerial parts	[12]
	*Pipturus albidus Hook. & Arn.*	leaves	[13]
Catechin	*Cecropia schreberiana* Miq.	leaves	[14]
Luteolin	*Urtica artichocaulis*	aerial parts	[12]
	*Urtica dioica*	leaves	[15]
Isorhamnetin	*Urtica dioica*	seeds	[12]
Kaempferol			
Quercetin	*Urtica artichocaulis*	aerial parts	[12]
	*Urtica cannabina* L.	fruits	[16]
	*Urtica dioica*	aerial parts	[15]
	*Boehmeria rugulosa* Wedd.	leaves	[17]
Luteolin-7-*O-β*-D-glucopyranoside	*Urtica laetevirens* Maxim.	aerial parts	[18]
Rutin	*Boehmeria nivea* L.	leaves	[19]
	*Boehmeria nivea*	roots	[20]
	*Urtica artichocaulis*	aerial parts	[12]
	*Urtica laetevirens*	aerial parts	[18]
Isovitexin	*Urtica cannabina*	fruits	[16]
	*Phenax angustifolius* Wedd.	leaves	[21]
Astragalin	Urtica cannabina	fruits	[16]
	Urtica dioica	seeds	[12]
Apigenin-7-*O*-glucoside	*Pilea microphylla* L.	leaves	[22]
Quercetin	*Boehmeria rugulosa*	leaves	[17]
	*Urtica artichocaulis*	aerial parts	[12]
	*Urtica cannabina*	fruits	[16]
	*Urtica dioica*	aerial parts	[15]
Quercetin-3-*O-*α-L-rhamnopyranoside	*Phenax angustifolius*	leaves	[21]

**Table 3:** Antiproliferative activity of *P. judaica* leaf extracts and selected chemotherapeutic agents after 24 h of incubation (MTT assay).

	IC_50_ [μg/mL]							
Extract	HEK-293	LN-229	NCI-H1563	MDA-MB-231	HepG2	769-P	HeLa	A-375
PJ-H	>300.00	115.62	133.12	122.92	107.86	128.51	112.45	113.68
PJ-M	202.34	74.18	121.13	141.29	102.17	144.12	63.21	93.06
PJ-E	139.42	21.27	18.97	19.54	49.57	27.82	11.82	14.09
PJ-W	253.35	93.68	87.69	126.83	133.41	142.78	146.67	188.78
Cisplatin	4.72 [23]	-	0.90 [24]	9.21 [25]	7.65 [26]	5.76 [27]	12.80 [28]	0.39 [29]
Temozolomide	-	87.84 [30]	-	-	-	-	-	-

**Table 4:** Cytotoxicity activity of *P. judaica* leaf extracts after 48 h of incubation (SRB assay).

	IC_50_ [μg/mL]							
Extract	HEK-293	LN-229	NCI-H1563	MDA-MB-231	HepG2	769-P	HeLa	A-375
PJ-H	270.62	132.78	108.16	139.17	81.89	107.99	115.59	53.51
PJ-M	>300.00	105.39	133.40	165.91	57.16	68.29	78.52	33.17
PJ-E	228.83	46.54	34.72	49.15	16.12	22.24	28.71	8.29
PJ-W	>300.00	108.97	92.51	123.69	83.46	96.56	168.03	112.94

## Data Availability

Data available on request.

## Supplementary Materials

The following supporting information can be downloaded at https://www.mdpi.com/article/10.3390/molecules30132739/s1, Table S1: LC-ESI-MS/MS analytical results of phenolic acids investigated in samples. Compounds confirmed by comparison with authentic standards. MRM transitions (precursor/fragment ion) selected for quantitative analysis are highlighted in bold for each compound; Table S2: Limit of detection (LOD), limit of quantification (LOQ), and calibration curve parameters for phenolic acids; Table S3: Summary of optimized parameters for the quantitative analysis of flavonoid compounds. MRM transitions (precursor/fragment ion) selected for quantitative analysis are highlighted in bold for each compound; Table S4: Analytical parameters of LC-MS/MS quantitative method for determination of flavonoid compounds.
