# Supplementary material for: Phytochemical Profile and Selective Anticancer Activity of Parietaria judaica L. Extracts
Source: Molecules. 2025 Jun 25;30(13):2739. doi: 10.3390/molecules30132739 (PMC12251272; doi:10.3390/molecules30132739)
Supplement: Supplementary file 1 [file molecules-30-02739-s001.zip › molecules-3651938-supplementary.pdf]

Table S1. LC-ESI-MS/MS analytical results of phenolic acids investigated in samples. Compounds confirmed by comparison with authentic standards. MRM transitions (precursor/fragment ion) selected for quantitative analysis are highlighted in bold for each compound.

Compound	Retention time [min]	[M-H]⁻ [*m/z*]	Fragment ions [*m/z*]	Colision energy [eV]
Gallic acid – SA PHL89198	4.82	**168.7**	**78.9**<br>124.9	-36<br>-14
3-O-caffeoylquinic acid (neochlorogenic acid) – SA PHL80504	6.66	**353**	**191**<br>178.9	-30<br>-30
Protocatechuic acid – SA PHL89766	7.43	**152.9**	**80.9**<br>107.8	-26<br>-38
5-caffeoylquinic acid (chlorogenic acid) – SA PHL89175	8.80<br>9.91	**352.9**	**190.8**<br>84.9	-24<br>-60
4-caffeoylquinic acid (cryptochlorogenic acid) – SA PHL10012	9.11	**353**	**173**<br>135	-30<br>-30
Gentisic acid – SA 78069	10.65	**352.9**	**80**<br>96.9	-110<br>-52
Caffeic acid – SA C0625	10.75	**178.7**	**88.9**<br>134.9	-46<br>-16
Syringic acid – SA PHL82659	10.90	**196.9**	**122.8**<br>181.9	-24<br>-12
Vanilic acid – SA PHL89819	10.90	**166.8**	**107.9**<br>123	-18<br>-12
p-Coumaric acid – SA 3200595	13.37	**162.8**	**93**<br>119	-44<br>-14
Ferulic acid – SA PHR1791	13.86	**192.8**	**133.9**<br>177.9	-16<br>-12
Rosmarinic acid – SA PHL89266	15.46	**358.7**	**132.6**<br>160.8	-44<br>-20
Salicylic acid – SA PHL89266	17.62	**136.9**	**75**<br>93	-48<br>-16

* SA – Sigma Aldrich.

Table S2. Limit of detection (LOD), limit of quantification (LOQ) and calibration curve parameters for phenolic acids.

Compound	LOD [ng/mL]	LOQ [ng/mL]	Equation of calibration curve	R^2	Linearity range [ng/mL]
Gallic acid	33.3	95	$y= 230\cdot x + 6.34\cdot 10^5$	0.9987	167-3300
3-*O*-caffeoylquinic acid (neochlorogenic acid)	20	40	$y= 300\cdot x + 2.39\cdot 10^3$	0.9996	40-10000
Protocatechuic acid	17	34	$y= 172\cdot x – 9.09\cdot 10^4$	0.9997	34-3470
5-caffeoylquinic acid (chlorogenic acid)	72	180	$y= 878\cdot x + 7.35\cdot 10^4$	0.9991	180-18000
4-caffeoylquinic acid (cryptochlorogenic acid)	20	40	$y= 215\cdot x + 1.05\cdot 10^3$	0.9979	40-4000
Gentisic acid	1.7	3.3	$y= 709\cdot x + 1.34\cdot 10^6$	0.9997	3.3-330
Caffeic acid	60	160	$y= 386\cdot x + 2.05\cdot 10^5$	0.999	175-3500
Syringic acid	167	666	$y= 12.9\cdot x + 1.32\cdot 10^4$	0.9993	666-11100
Vanillic acid	100	250	$y= 10.1\cdot x + 2.42\cdot 10^4$	0.9997	330-33000
p-Coumaric acid	7.3	18.1	$y= 387\cdot x + 1.22\cdot 10^5$	0.9996	18.1-1820
Ferulic acid	17.4	34.7	$y= 1.82\cdot x - 786$	0.9994	69.4-11600
Rosmarinic acid	7.1	17.9	$y= 255\cdot x + 2.56\cdot 10^4$	0.9994	17.9-7140
Salicylic acid	3.3	16.5	$y= 567\cdot x + 1.54\cdot 10^6$	0.9989	16.5-1650

Table S3. Summary of optimized parameters for the quantitative analysis of flavonoid compounds. MRM transitions (precursor/fragment ion) selected for quantitative analysis are highlighted in bold for each compound.

Compound	Retention time [min]	$[M-H]^-$ [*m*/*z*]	Fragment ions [*m*/*z*]	Colision energy [eV]
		**Flavonoid aglycones**		
Catechin – SA PHL89172	9.29	**288.8**	**244.9**	-16
			109	-32

EGCG – SA PHR1333	10.85	**168.7**	**78.9**<br>124.9	-30<br>-30
Dihydromyricetin – SA PHL80431	11.73	**331**	**315.9**<br>151	-30<br>-30
Myricetin – SA PHL89252	16.57	**316.7**	**136.9**<br>150.9	-32<br>-26
Eriodictyol – SA PHL80444	17.71	**286.7**	**134.9**<br>150.9	-32<br>-18
Luteolin – SA PHL89245	17.65	**284.7**	**132.9**<br>150.9	-38<br>-26
Quercetin – SA PHR1488	17.76	**300.7**	150.9<br>178.8	-26<br>-20
3-*O*-Methylquercetin – SA 90081	17.94	**314.7**	**299.8**<br>270.8	-18<br>-26
Apigenin – SA PHL89159	18.43	**268.8**	**117**<br>106.8	-44<br>-34
Kaempferol – SA PHL89235	16.64	**284.7**	**116.8**<br>93	-46<br>-52
Isorhamnetin – SA PHL89314	18.73	**314.7**	**299.7**<br>150.9	-20<br>-30
		**Flavonoid glycosides**		
Luteolin 3′,7′-diglucoside – SA PHL89246	11.01	**609.1**	285<br>447	-50<br>-32
Quercetin -3-*O*-rutinoside (Rutin) – SA PHL83535	11.69	**608.7**	**299.6**<br>270.9	-46<br>-60
Apigenin – 6-*C*-glucoside (Isovitexin) – SA PHL89233	11.98	**430.8**	**310.9**<br>340.9	-28<br>-26
Luteolin-7-*O*-glucoside (Luteoloside) – SA 1370837	12.44	**446.8**	**284.8**<br>132.9	-30<br>-78
Quercetin -3-*O*-glucoside (Isoquercetin) – SA 17793	12.58	**462.7**	**299.7**<br>270.7	-30<br>-44
Eriodictyol-7-*O*-glucopyranoside – SA 94258	12.65	**448.8**	**286.9**<br>134.9	-24<br>-48
Kaempferol – 3-*O*-rutinoside (Nicotiflorin) – SA PHL80700	12.83	**592.7**	**284.8**<br>226.7	-38<br>-68
Isorhamnetin-3-*O*-rutinoside	13.00	**622.8**	**314.9**<br>298.8	-40<br>-52

(Narcissoside) – SA PHL83337				
Kaempferol – 3-*O*-glucoside (Astragalin) – SA PHL89237	14.25	**446.7**	**226.8**<br>254.8	-54<br>-40
Isorhamnetin-3-glucoside – SA 17794	14.37	**476.8**	**313.9**<br>270.9	-30<br>-44
Quercetin 3-*O*-rhamnoside (Quercitrin) – SA PHL89346	14.45	**446.7**	**299.7**<br>270.7	-30<br>-40
Apigenin 7-*O*-glucoside (Apigetrin, Cosmosiin) – SA PHL89160	14.55	**430.7**	**267.7**<br>116.9	-38<br>-84
Naringenin 7-*O*-glucoside – SA 1354954	14.82	**432.7**	**270.8**<br>118.9	-22<br>-64

* SA – Sigma Aldrich.

Table S4. Analytical parameters of LC-MS/MS quantitative method for determination of flavonoid compounds.

**Compound**	**LOD [ng/mL]**	**LOQ [ng/ mL]**	**Equation of calibration curve**	R^2	**Linearity range [ng/ mL]**
		**Flavonoid aglycones**			
Catechin	200	300	y= 55.2·x + $1.78 \cdot 10^4$	0.9982	300-6600
EGCG	1000	1500	y= 426·x + $7.62 \cdot 10^4$	0.9986	1500-18500
Dihydromyricetin	15	25	y= 129·x - $1.52 \cdot 10^3$	0.9989	25-3000
Myricetin	3300	6600	y= 72.6·x + $3.61 \cdot 10^5$	0.9995	6600-66000
Eriodictyol	10	12	y= 403·x + $1.01 \cdot 10^5$	0.9982	12-5000
Luteolin	25	50	y= $1.37 \cdot 10^3$·x + $5.58 \cdot 10^4$	0.9989	50-5000
Quercetin	5	15	y= 136·x + $3.58 \cdot 10^4$	0.9987	15-3000
3-*O*-Methylquercetin	5	15	y= 476·x + $4.24 \cdot 10^4$	0.9984	15-3500
Apigenin	5	15	y= 523·x + $7.73 \cdot 10^4$	0.9990	15-5000
Kaempferol	20	30	y= 159·x + $3.24 \cdot 10^4$	0.9989	30-20000
Isorhamnetin	15	30	y= 411·x + $6.07 \cdot 10^4$	0.9991	50-60000
		**Flavonoid glycosides**			
Luteolin 3′,7′-diglucoside	250	500	y= 12.3·x – $4.61 \cdot 10^3$	0.9988	1000-20000
Quercetin -3-*O*-rutinoside (Rutin)	115	230	y= 76.3·x + $8.81 \cdot 10^4$	0.9983	2000-50000

Apigenin – 6-*C*-glucoside (Isovitexin)	100	200	$y = 86.4 \cdot x + 4.37 \cdot 10^4$	0.9990	1200-30000
Luteolin-7-*O*-glucoside (Luteoloside)	50	100	$y = 128 \cdot x + 8.11 \cdot 10^3$	0.9979	300-20000
Quercetin -3-*O*-glucoside (Isoquercetin)	150	300	$y = 80.5 \cdot x + 1.61 \cdot 10^5$	0.9982	2000-25000
Eriodictyol-7-*O*-glucopyranoside	100	250	$y = 102 \cdot x + 8.84 \cdot 10^4$	0.9990	1670-50000
Kaempferol – 3-*O*-rutinoside (Nicotiflorin)	60	120	$y = 81.8 \cdot x + 1.54 \cdot 10^4$	0.9989	120-50000
Isorhamnetin-3-*O*-rutinoside (Narcissoside)	100	200	$y = 145 \cdot x – 3.11 \cdot 10^4$	0.9992	200-2500
Kaempferol – 3-*O*-glucoside (Astragalin)	100	200	$y = 149 \cdot x + 1.37 \cdot 10^5$	0.9980	1200-24000
Isorhamnetin-3-glucoside	100	300	$y = 102 \cdot x + 8.84 \cdot 10^4$	0.9985	2000-20000
Quercetin 3-*O*-rhamnoside (Quercitrin)	50	100	$y = 102 \cdot x + 4.64 \cdot 10^4$	0.9981	1000-25000
Apigenin 7-*O*-glucoside (Apigetrin, Cosmosiin)	100	250	$y = 85.1 \cdot x + 1.15 \cdot 10^5$	0.9989	1500-25000
Naringenin 7-*O*-glucoside	100	250	$y = 84.8 \cdot x + 4.83 \cdot 10^3$	0.9987	250-25000
